# Phytoestrogen genistein hinders ovarian oxidative damage and apoptotic cell death-induced by ionizing radiation: co-operative role of ER-β, TGF-β, and FOXL-2

**DOI:** 10.1038/s41598-020-70309-2

**Published:** 2020-08-11

**Authors:** Yasmin Hamdy Haddad, Riham S. Said, Rehab Kamel, Engy M. El Morsy, Ebtehal El-Demerdash

**Affiliations:** 1 Central Administration of Pharmaceutical Affairs, Cairo, Egypt; 2 Department of Drug Radiation Research, National Center for Radiation Research and Technology, Atomic Energy Authority, Cairo, Egypt; 3 Department of Pharmacology and Toxicology, Faculty of Pharmacy, Helwan University, Cairo, Egypt; 4 Department of Pharmacology and Toxicology, Faculty of Pharmacy, Ain Shams University, Cairo, Egypt

**Keywords:** Drug discovery, Diseases, Endocrinology

## Abstract

Radiotherapy is a well-known cause of premature ovarian failure (POF). Therefore, we investigated the molecular influence of genistein (GEN) on the ovarian reserve of rats exposed to ϒ-radiation. Female Sprague Dawley rats were exposed to a 3.2 Gy γ-radiation to induce POF and/or treated with either GEN (5 mg/kg, i.p.) or Ethinyl estradiol (E2; 0.1 mg/kg, s.c.), once daily for 10 days. GEN was able to conserve primordial follicles stock and population of growing follicles accompanied with reduction in atretic follicles. GEN restored the circulating estradiol and anti-Müllerian hormone levels which were diminished after irradiation. GEN has potent antioxidant activity against radiation-mediated oxidative stress through upregulating endogenous glutathione levels and glutathione peroxidase activity. Mechanistically, GEN inhibited the intrinsic pathway of apoptosis by repressing Bax expression and augmenting Bcl-2 expression resulted in reduced Bax/Bcl-2 ratio with subsequent reduction in cytochrome c and caspase 3 expression. These promising effects of GEN are associated with improving granulosa cells proliferation. On the molecular basis, GEN reversed ovarian apoptosis through up-regulation of ER-β and FOXL-2 with downregulation of TGF-β expression, therefore inhibiting transition of primordial follicles to more growing follicles. GEN may constitute a novel therapeutic modality for safeguarding ovarian function of females’ cancer survivors.

## Introduction

Radiotherapy is one of the most important strategies in cancer treatment. Nearly 80% of cancer patients are subjected to radiotherapy at some stage or other in their treatment regimen^1^. Seriously, radiotherapy results in premature ovarian failure (POF) and infertility, mostly due to the absence of follicles, or the inability of the remaining follicles to respond to gonadotropin stimulation leading subsequently to oocyte loss and ovarian atrophy with reduced follicle stores^2^.

Radiotherapy depends on the generation of reactive oxygen species (ROS) in cancer cells as a result of water radiolysis leading to induction of oxidative stress and diminution of antioxidant defense mechanisms and within this process, healthy tissues are also damaged^3^. Remarkably, the disorder between free radicals and oxidative radicals is believed to be a potential etiology of POF^4^. Moreover, germ cells seem to be much more susceptible to oxidative stress induced by radiotherapy than somatic cells^5^. Seriously, ROS generated by ionizing radiation are capable of inducing tissue apoptosis by direct and indirect pathways leading to oxidative damage to cellular macromolecules (mainly DNA, proteins and lipids)^6^. Consequently, ROS activate the intrinsic mitochondrial pathway of apoptosis via activating tumor suppressor protein (p53) which in turn increases mitochondrial membrane permeability, leading to cytochrome c release and activation of caspases^7^. Curiously, apoptosis was identified as the mechanism responsible for oocyte loss caused by cancer treatment^8^ and is further induced by radiation^9^.

Besides oxidative stress and apoptosis, it was reported that fibrotic and pro-inflammatory cytokines such as transforming growth factor (TGF)-β play an important role in tissue injury induced by radiation^10^. Remarkably, many members of the TGF-β superfamily promote ovarian development through activating the transition of primary follicles into the pre-antral and antral stages leading to follicular depletion^11^. Thus, to preserve female fertility and overcome the deleterious effects of radiation on ovarian function, it is necessary to discover new effective radioprotective agents to limit oxidative stress, apoptosis, and TGF-β signaling.

Soybeans products contain high amounts of isoflavones known as soy phytoestrogens which act as natural selective estrogen receptor modulators (SERMs)^12^. The most prominent phytoestrogen in soybean is genistein (GEN)^13^, which shows estrogenic properties through its ability to bind to estrogen receptor beta (ER-β)^14^. GEN has different pharmacological properties due to its chemoprotective activity against cancers and cardiovascular diseases^15^. GEN was also reported to protect against acute myelotoxicity^16^, intestinal^17^, lung^18,19^, and testicular injuries-induced by radiation^20^. The radioprotective effects of GEN were attributed to its antioxidant, anti-apoptotic, anti-inflammatory and anti-fibrotic activities^18,21^. Concerning its effects on the ovaries, a previous report confirmed the protective effect of GEN against ovarian carcinogenesis^22^. Also, GEN slowed down follicular development, considerably improving the ovarian follicular stock and extending the ovarian lifespan^23^. In this context, GEN was documented to delay ovarian ageing and prolong ovarian reproductive life^21^, besides its protective effect against chemotherapy-induced ovarian toxicity^24^.

Based on this background, we investigated the potential modulatory effect of GEN on radiation-induced POF in rats and studied the underlying molecular mechanism. Within this objective, we investigated the effect of GEN on folliculogenesis, oxidative stress, apoptosis, proliferation markers as well as its impact on ER-β, forkhead box L2 protein (FOXL2), and TGF-β expression. Additionally, we compared the effects of GEN to Ethinyl estradiol (E2) to determine whether the ovarian radioprotective effects of GEN are correlated or not to its ER-β binding.

## Results

### Body and ovarian weights changes

At the end of the study, we observed that animals subjected to ϒ-radiation acquired less body weight as compared to the control group (Table 1). We also compared the ovarian weights after normalizing them to 100 g body weight. Ovarian weight was markedly decreased in the irradiated group by 20% as compared to the control group while treatment with GEN or E2 considerably prohibited the influences of irradiation and kept body and ovarian weights almost matching those of the control group. Rats treated with GEN alone showed no significant difference in body and ovarian weights when compared to the control group (Table 1).

**Table 1:** Effect of genistein (GEN) or Ethinyl estradiol (E2) treatment on body and ovarian weight loss induced by whole-body irradiation (IR).

Groups	Changes in total body weight (g)	Relative ovarian weight (g/100 g body weight)
Control	18.46 ± 6.10	0.10 ± 0.01
IR	7.42 ± 2.32^a^	0.08 ± 0.02^a^
GEN	20.68 ± 6.28^b^	0.10 ± 0.02^b^
GEN/IR	18.78 ± 6.17^b^	0.10 ± 0.02^b^
E2/IR	14.80 ± 4.63^b^	0.11 ± 0.02^b^

Data are expressed as mean ± SD, (N = 8).

Statistical analysis was done using ANOVA followed by Tukey–Kramer as a post hoc test.

^a,b^Statistically significant difference to control or radiation group, respectively at p < 0.05.

### Circulating hormone levels

**Figure 1:**
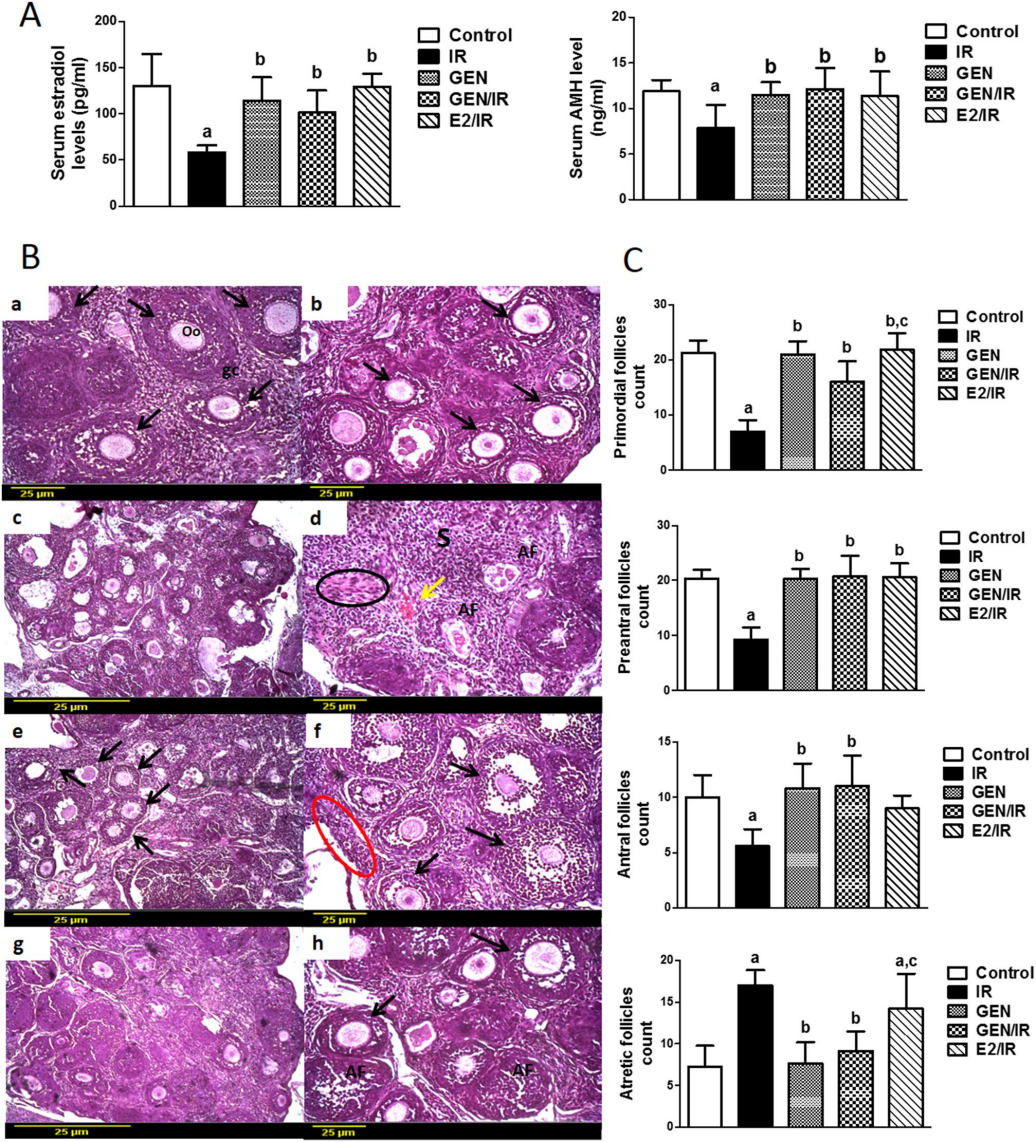
Circulating hormone levels and ovarian follicles' assembly. (**A**) Changes in serum levels of estradiol and Anti- Müllerian hormone (AMH) in female rats subjected to γ-radiation (IR) and/or treated with genistein (GEN) or Ethinyl estradiol (E2). Values are given as mean ± SD. (**B**) Photomicrographs of ovarian sections stained with hematoxylin and eosin. Ovarian sections from control (a, ×20) and from genistein alone (b, ×20) treated rats have normal structure with healthy follicles at different stages (black arrows), integral oocytes (Oo), and granulosa cells (gc). Ovarian sections from irradiated rats (c, ×10; d, ×20) exhibit numerous atretic follicles (AF), severe hemorrhage (yellow arrow), and fibrosis (black circle). Ovarian sections from irradiated rats treated with genistein (e, ×10; f, ×20) show normal structure with different types of follicles (black arrows) including many primordial follicles (red circle). Ovarian sections of irradiated rats treated with Ethinyl estradiol (g, ×10; h, ×20) show some healthy follicles (black arrows) with increased number of atretic follicles (AF). Scale bar, 25 µm. *AF* atretic follicles, *gc* granulosa cell, *Oo* oocyte, *S* stroma. (**C**) Morphometric analysis of primordial, preantral, antral and atretic follicle population. Columns represent mean ± SD of different five rat ovaries. “a, b, or c” indicates statistically significant difference to control, irradiated (IR) or genistein/irradiated (GEN/IR) group, respectively at p < 0.05 using one-way ANOVA followed by Tukey–Kramer as a post-hoc test.

Exposure of female rats to γ-radiation dramatically decreased both serum estradiol and AMH levels by 55% and 34%, respectively, when compared with those of the control group. In contrast, treatment of irradiated rats with GEN or E2 showed a significant elevation in serum estradiol and AMH levels keeping them similar to those in the control group. Treatment of female rats with GEN alone didn't alter serum hormone levels as compared to the control group (Fig. 1A).

### Ovarian histology

Microscopic examination of ovarian sections from control and GEN alone groups showed normal histological structure identified by the presence of growing follicles at various developmental stages (primordial, pre-antral, antral and graffian follicles), intact layers of granulosa cells, as well as normal oocytes and corpus luteum formation (Fig. 1B; a, b). Contrariwise, sections of irradiated ovaries revealed the predominance of stromal cells with diffused hemorrhage, interstitial hyperplasia, and marked fibrosis. Irradiated sections also revealed depleted stock of primordial as well as growing follicles with abundant atretic follicles containing atrophic oocytes (Fig. 1B; c, d). Interestingly, administration of GEN (Fig. 1B; e, f) or E2 (Fig. 1B; g, h) protected the ovarian tissue from hemorrhage and fibrosis caused by radiation and prohibited loss of primordial and developing follicles.

Then, the population of primordial, growing, and atretic follicles of different cohorts was determined. Morphometric analysis proved that γ-radiation caused a great reduction in the count of almost all types of healthy follicles. Radiation decreased the primordial follicles pool, preantral and antral follicles by 67%, 55%, and 47%, respectively, accompanied by a drastic increase in atretic follicles by 134% as compared to control group (Fig. 1C). Contrariwise, treatment with GEN markedly increased the population of primordial, preantral and antral follicles and significantly decreased atretic follicles count as compared to the irradiated rats. However, E2 treatment significantly increased the primordial and preantral follicle populations without affecting the antral or atretic follicles count as compared to the irradiated group. Moreover, compared to GEN treated group, E2 administration significantly increased the primordial follicles population. Therefore, the results of the morphometric analysis revealed that GEN and E2 treatment have different influence on ovarian folliculogenesis. While GEN administration preserves all types of ovarian follicles, E2 greatly increased primordial follicles stock than hindering follicular atresia.

### Oxidative stress markers

Oxidative stress induced by gamma-radiation in rat ovaries was assessed by measuring reduced GSH levels as well as GPx activity. As shown in Table 2, ϒ-radiation induced a significant reduction in both ovarian GSH level and GPx activity by 57% and 30%, respectively as compared to the control group. However, the treatment of irradiated rats with GEN or E2 almost resumed normal levels of GSH level and GPx activity. Treatment with GEN alone did not show any marked difference in neither GSH level nor GPx activity.

**Table 2:** Effect of genistein (GEN) or Ethinyl estradiol (E2) administration on oxidative stress markers in rats exposed to a single dose whole-body irradiation (IR).

Groups	Reduced GSH level (μg/mg tissue)	Glutathione peroxidase activity (U/g tissue)
Control	56.60 ± 13.86	4.07 ± 0.54
IR	24.09 ± 8.09^a^	2.87 ± 0.08^a^
GEN	68.07 ± 10.64^b^	4.86 ± 0.61^b^
GEN/IR	67.77 ± 7.98^b^	4.89 ± 0.53^b^
E2/IR	60.29 ± 7.63^b^	5.91 ± 1.65^a,b^

Data expressed as Mean ± SD (N = 8).

Statistical analysis was done using ANOVA followed by Tukey–Kramer as a post hoc test.

^a,b^Statistically significant difference to control or radiation group, respectively at p < 0.05.

### Apoptotic markers

**Figure 2:**
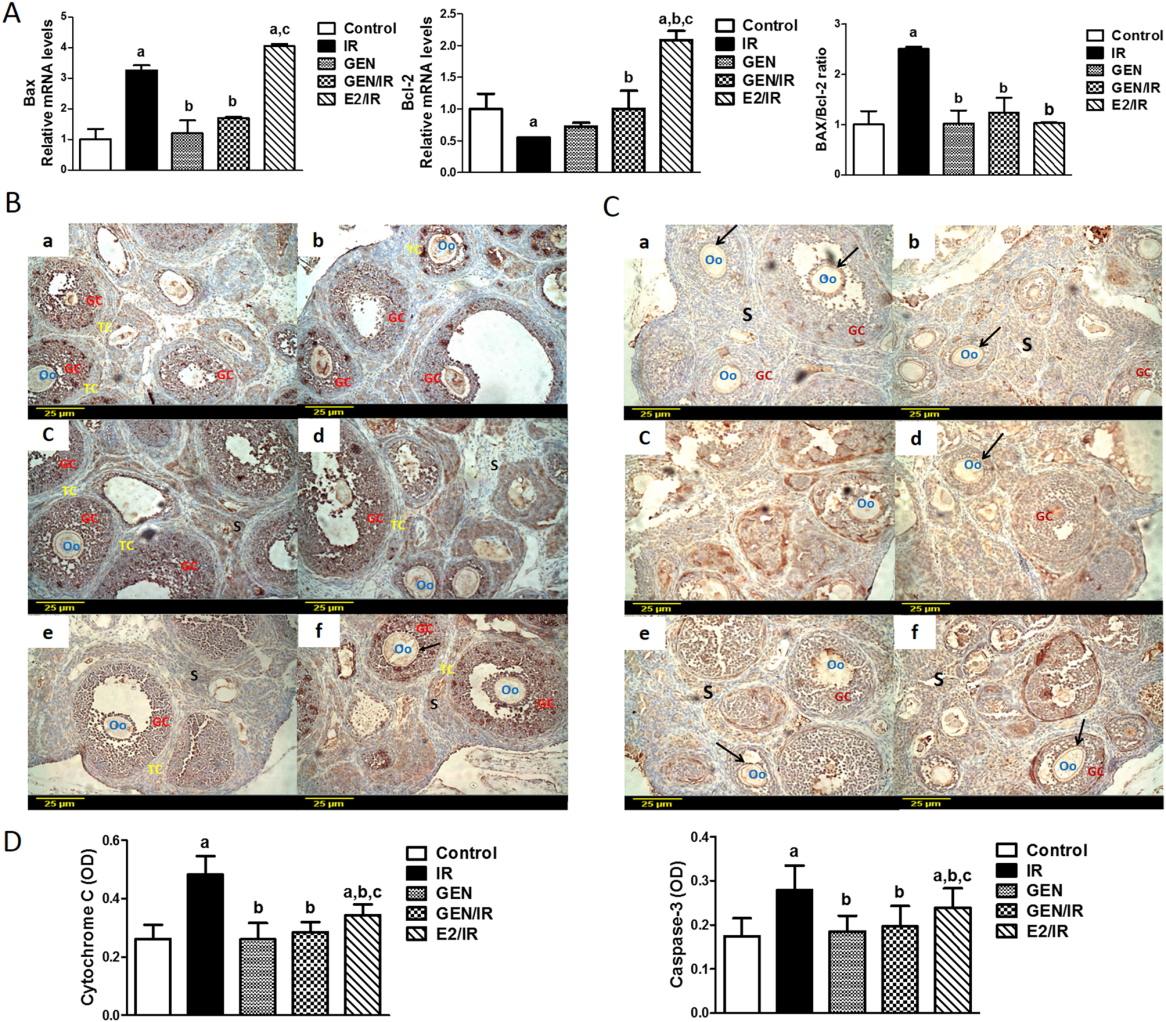
Genistein attenuates the intrinsic pathway of apoptosis-induced by ϒ-radiation. (**A**) Real-time PCR analysis of ovarian Bax and Bcl-2 mRNA expression. Data are represented as fold changes relative to control values. Columns represent mean ± SD. “a, b or c” indicates statistically significant difference from control, irradiated (IR) or genistein/irradiated (GEN/IR) group, respectively at p < 0.05 using one-way ANOVA followed by Tukey–Kramer as a post-hoc test. Immunohistochemical localization of ovarian cytochrome C (**B**) and caspase 3 (**C**) in control group (a), genistein alone treated group (b), irradiated group (c&d), irradiated/genistein treated group (e), and irradiated/Ethinyl estradiol treated group (f), ×20. Scale bar, 25 µm. *Oo* oocyte, *GC* granulosa cells, *S* stromal cells; black arrows point to Zona pellucida cells. **(D)** Quantification of immunohistochemical staining represented as optical density (OD); averaged across 7 high-power fields. Columns represent mean ± SD. “a, b or c” indicates statistically significant difference to control, irradiated (IR) or genistein/irradiated (GEN/IR) group, respectively at p < 0.05 using one-way ANOVA followed by Tukey–Kramer as a post-hoc test.

The effects of radiation on the mitochondrial-dependent apoptotic pathway were examined by analysis of Bax and Bcl-2 gene expression by quantitative real-time PCR. The results revealed that γ-radiation significantly increased Bax mRNA expression by 3 folds while decreased the transcript levels of anti-apoptotic Bcl-2 expression by 0.5 fold compared to the control group. By calculating Bax to Bcl-2 ratio, irradiation significantly increased Bax/Bcl-2 ratio by 2.5 folds as compared to the control values (Fig. 2A). Nevertheless, treatment of irradiated rats with GEN significantly decreased Bax expression by 48% while increased Bcl-2 expression by 82% leading subsequently to a significant reduction in Bax/Bcl-2 ratio by 50% as compared to the irradiated group. Alternatively, treatment of irradiated rats with E2 did not show any significant difference in Bax mRNA expression as compared to irradiated group while significantly increased Bcl-2 mRNA expression levels which resulted in reduction in Bax/Bcl-2 ratio which was upregulated by radiation. Obviously, as compared to the GEN group, E2 treated rats had higher Bax and Bcl2 mRNA expression with similar Bax/Bcl-2 ratio. GEN alone treated rats didn’t show any significant difference in Bax or Bcl-2 expression as compared to control values (Fig. 2A).

We also determined the protein expression of cytochrome c and caspase 3 by immunohistochemical staining of ovarian sections. Cytochrome c and caspase 3 were rarely expressed in ovarian theca and granulosa cells of the control rats (Fig. 2B,C; a). Ovarian sections from rats treated with GEN alone showed similar expression of both cytochrome c and caspase 3 as that seen in the control group (Fig. 2B,C; b). However, the protein expression of both apoptotic proteins was largely upregulated by γ-radiation, this increased expression was reflected by the high intensity of the brown stain of cytochrome c and caspase 3 proteins which were expressed mainly in granulosa and theca cells of atretic follicles as well as interstitial cells (Fig. 2B,C; c, d). Treatment of irradiated rats with GEN or E2 inhibited the marked elevation in the expression of cytochrome c and caspase 3 in ovarian granulosa and theca cells induced upon radiation exposure (Fig. 2B,C; e, f).

Quantification of cytochrome c and caspase 3 immunoreactivity showed that irradiation significantly increased the optical densities of immunopositive cells by 84% and 60%, respectively, as compared to the control group (Fig. 2D). In contrast, GEN or E2 treatment significantly reduced the marked elevation in the optical densities of both cytochrome c and caspase 3 induced by irradiation. However, GEN treatment significantly decreased cytochrome c and caspase 3 optical densities by 25% and 23.5%, respectively, as compared to the E2 group suggesting the potent anti-apoptotic effects of GEN over E2.

### Proliferation marker PCNA

**Figure 3:**
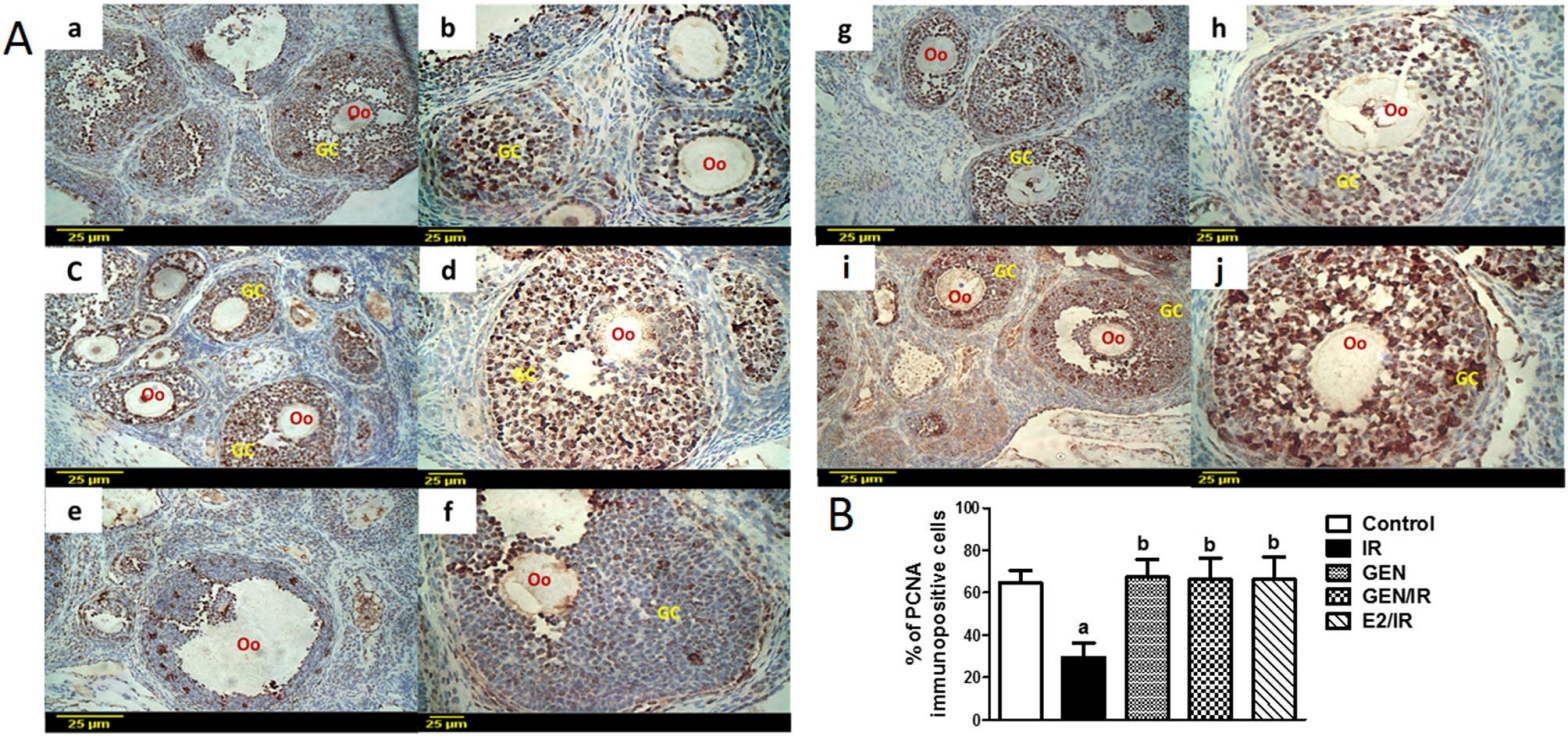
Effect of genistein or Ethinyl estradiol treatment on PCNA expression in ovarian follicles of irradiated rats. (**A**) Immunohistochemical localization of PCNA. Ovarian sections from control (a, ×20, b, ×40) and from genistein alone (c, ×20, d, ×40) treated rat's exhibit high level of PCNA expression. Ovarian sections from irradiated rats (e, ×20, f, ×40) exhibit negligible expression of PCNA. Ovarian sections from irradiated rats treated with genistein (g, ×20, h, ×40) exhibit PCNA expression similar to that of control ovaries. Ovarian sections from irradiated rats treated with Ethinyl estradiol (i, ×20, j, ×40) exhibit elevated PCNA expression. Scale bar, 25 µm; *Oo* oocyte, *GC* granulosa cells. (**B**) Quantitative immunohistochemical staining of PCNA represented as the percentage of immunopositive cells to the total number of granulosa cells across 7 high power fields (×40) for each rat section. Values are given as mean ± SD. “a or b” indicates statistically significant difference to control or radiation group, respectively at p < 0.05 using one-way ANOVA followed by Tukey–Kramer as a post-hoc test.

Granulosa cells' proliferation was evaluated through immunohistochemical detection of PCNA (Fig. 3). Ovarian sections obtained from the control (Fig. 3A; a, b) and GEN alone (Fig. 3A; c, d) treated rats showed that most oocytes and granulosa cells of growing follicles were positively stained. In contrast, ovarian sections from irradiated rats showed that granulosa cells of most follicles including the most developed ones were negatively stained (Fig. 3A; e, f) which is further confirmed quantitatively by counting the number of PCNA positive cells in seven high power fields (Fig. 3B). In this context, γ-radiation significantly decreased the percentage of PCNA immunopositive cells by 55% as compared to the control ovaries. On the other hand, ovarian sections from irradiated rats treated with either GEN (Fig. 3A; g, h) or E2 (Fig. 3A; i, j) revealed that 66% of PCNA labeled granulosa cells of healthy preantral and antral follicles were positively-stained (Fig. 3B).

### Ovarian mRNA expression of ER-β, FOXL2, and TGF-β

**Figure 4:**
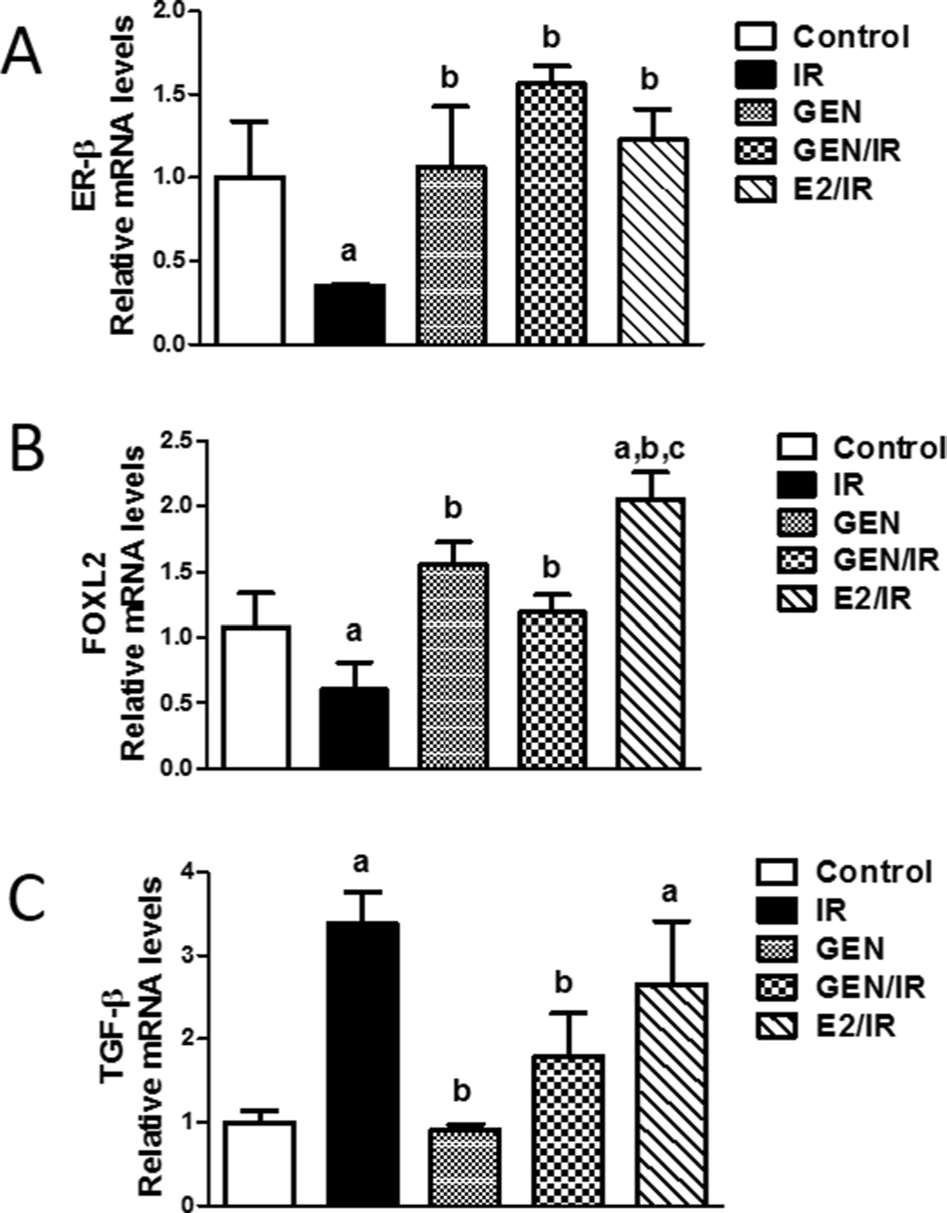
Quantitative real-time PCR of ER-Beta, FOXL2 and TGF-β mRNA levels in ovaries of irradiated, genistein or ethinyl estradiol treated rats. All mRNA was normalized to mRNA expression of GAPDH. Data is represented as fold changes relative to control values. Columns represent mean ± SD. “a, b or c” indicates statistically significant difference to control, irradiation (IR) or genistein/irradiated (GEN/IR) group, respectively at p < 0.05 using one-way ANOVA followed by Tukey–Kramer as a post-hoc test.

Ionizing radiation exposure led to a significant decrease in both ER-β and FOXL2 transcription levels by 0.35 fold and 0.55 fold, respectively (Fig. 4A,B), accompanied with a significant increase in TGF-β mRNA expression by 3 folds as compared to control group (Fig. 4C). Conversely, the treatment of irradiated rats with GEN or E2 significantly increased the mRNA levels of ER-β and FOXL2 as compared to the irradiated group. Moreover, treatment of irradiated rats with GEN markedly decreased TGF-β mRNA expression by 47% when compared to the irradiated group. However, the E2 administration could not ameliorate the increased TGF-β expression induced by γ-radiation. Obviously, the increased mRNA levels of FOXL-2 in the irradiated group treated with E2 were significantly higher than those of the control and GEN treated values. These findings illustrate that although both GEN and E2 administration upregulated ER-β and FOXL2 expression levels, E2 was not able to downregulate the higher TGF-β expression-triggered by radiation.

## Discussion

POF is one of the deleterious complications of therapeutic radiation to the whole body or pelvis ^25^, and therefore, new strategies for management of POF are absolutely required. Besides the anticancer effects of GEN, it exerts radioprotective effects on several organs such as liver, intestine, and testis^20,26,27^. Further, GEN attenuated cyclophosphamide-induced ovarian toxicity^24^. Hence, the current study investigated the protective effect of GEN against radiation-induced ovarian toxicity in rats through estimating the folliculogenesis process, follicular proliferation, ovarian hormones levels, mitochondrial apoptotic signaling pathway, as well as GEN molecular targets. Our findings confirmed that GEN administration during radiotherapy preserved the ovarian function as evidenced by the following: (1) preserving ovarian architecture and maintaining follicular stock, (2) augmenting estradiol and AMH levels, (3) boosting endogenous antioxidant defense, (4) hindering mitochondrial pathway of apoptosis through downregulating Bax/Bcl-2 ratio besides cytochrome c and caspse-3 expression, (5) enhancing granulosa cells proliferation, (6) upregulating ER-β and FOXL-2 expression while downregulating TGF-β expression. Together, our study stated for the first time the protective role of GEN against radiation-induced POF.

Comparison of ovarian reserve markers between control and irradiated rats showed significant impairment in the size of the ovaries with a drastic loss of primordial, preantral, and antral follicles population after 4 days of radiation exposure. These observations were also reported by previous studies^28^. Because the pool of primordial follicles is believed to be finite, the destruction of primordial follicles accelerates the onset of POF and leads very rapidly to female sterility^29^. Besides follicles loss and in consistent with previous studies^30^, an acceleration of follicular atresia occurred after irradiation. Interestingly, the treatment of irradiated rats with GEN markedly preserves the primordial and growing follicles population and inhibits follicular atresia reflecting the ovarian radioprotective effect of GEN. These data are consistent with previous findings^23^ supposing that GEN increased ovarian follicular reserve in early aged rats by slowing down the development of primordial follicles to growing follicles and by inhibiting follicular atresia thus prolonging the ovarian lifespan. However, in comparison to the GEN/IR group, E2 treated group showed a significant increase in primordial follicles counts suggesting the more inhibitory effect of E2 on primordial follicles transition than GEN. Moreover, E2 treated rats did not show any significant difference in the antral and atretic follicles population when compared to the irradiated ones. Therefore, histological and morphometric analysis data suggest that GEN is more able to preserve ovarian follicular stock after radiation exposure than E2 treatment.

Subsequently, diminished ovarian reserve and folliculogenesis impairment induced by γ-radiation leads to disordered hormone secretion. The current experiment showed a significant reduction in serum estradiol four days after γ-radiation which could be attributed to the profound toxicity to the ovarian reserve^31^. Conversely, diminished estradiol secretion could enhance the enrolment of primordial follicles into growing stages resulting in the depletion of primordial follicles stock and eventually POF^32^. In addition, the serum levels of AMH, a putative marker of ovarian reserve^33^ was also decreased in the present study. Remarkably, antral follicles count together with AMH levels are the most sensitive measures for quantifying damage to the ovaries^34^. One of the roles of AMH is to inhibit primordial follicle activation, which slows the rate at which the ovarian reserve is depleted^35^. Therefore, we suggest that primordial follicle depletion by γ-radiation can arise through excessive primordial follicles recruitment due to undetectable levels of AMH after irradiation damage of growing follicles. Moreover, AMH was reported as a regulator of follicular atresia and its expression is negligible in atretic follicles^36^. Interestingly, treatment with either GEN or E2 remarkably elevated serum estradiol and AMH levels which supports the previous findings revealing that GEN restored estradiol and AMH levels in cyclophosphamide treated rats^24^. The next aim of this study was to explore the molecular mechanisms underlying these potential radioprotective effects of GEN against ovarian follicles insufficiency.

Ionizing radiation induces cell damage by inducing metabolic oxidative stress through the formation of free radicals^37,38^ and consequently damage macromolecules resulting in the degeneration of tissue function^39^. The current study revealed a significant decrease in reduced GSH levels and GPx activity in ovarian tissues four days post-irradiation. Subsequently, GSH depletion could trigger granulosa cell apoptosis in adult rat ovaries^40^. In contrast, as GEN is an antioxidant, it reduced the toxic effects associated with oxidative injury by augmenting the antioxidant effects. Our results are in consistent with previous studies confirmed the antioxidant role of GEN in attenuating ovarian injury induced by chemotherapy or ischemia/reperfusion injury^24,41^. Therefore, GEN mitigated radiation-induced oxidative stress in a manner comparable to that observed in E2.

One of the critical pathways triggered by γ-radiation is the apoptotic pathway that could be resulted from DNA damage and oxidative stress. The intrinsic pathway is activated at the mitochondrial level, which subsequently triggers follicular apoptosis by caspases activation^42^. The follicular apoptosis is regulated both by pro-apoptotic factors (e.g., Bax) and anti-apoptotic factors (e.g., Bcl-2 and anti-oxidative agents)^43^. Proapoptotic factors contribute to disrupting the mitochondrial membrane integrity and the release of cytochrome c into the cytosol, which further activates caspase-9 and consequently other caspases^44^. On the other hand, the anti-apoptotic Bcl-2 proteins inhibit the oligomerization of the effector proteins Bax^45^. In the present study, quantitative PCR demonstrated that γ-radiation significantly upregulates pro-apoptotic Bax gene expression and conversely downregulates the anti-apoptotic Bcl-2 gene expression. Hence, the skewed Bax/Bcl2 ratio increased upon radiation exposure thereby activates the intrinsic apoptotic pathway. In addition, immunohistochemistry assessment revealed increased protein expression of cytochrome c and caspase-3 in the ovaries following radiation exposure which is considered as a terminal event prior to cell death^46^.

GEN treatment switched off the apoptotic machinery induced by irradiation through enhancing the expression of Bcl-2, diminishing the expression of Bax, and decreasing the ratio of Bax to Bcl-2, resulted in down-regulation of cytochrome c and caspase-3 protein expressions caused by irradiation. Although E2 treatment significantly improved Bax/Bcl2 ratio, cytochrome c, and caspase-3 protein expression, it increased Bcl-2 gene expression without affecting Bax expression compared to the irradiated group. Moreover, the irradiated group treated with GEN showed significantly lower expression of cytochrome c and caspase-3 proteins as compared to that treated with E2. Therefore, these findings confirmed that GEN overwhelmed E2 in improving the apoptotic events occurring in the ovary due to γ-radiation. These results reinforce the previous report stating that GEN improved ovarian function in the polycystic ovary model by reducing cell apoptosis^47^. Together, these results indicate that the radioprotective effect of GEN over E2 in attenuating ovarian injury could be through its antioxidant properties and modulation of the mitochondrial apoptotic pathways.

Besides the apoptosis pathway, if the cell is exposed to stress with ionizing radiation and DNA damage occurs, p21, an inhibitor of cyclin-dependent kinases, will be activated in a p53-dependent manner. Subsequently, the cell cycle is stopped at the G1 control point, and thus, PCNA-dependent DNA replication is inhibited^48^. PCNA is a sensitive marker of early events of granulosa cell proliferation where its expression increases significantly in granulosa and theca cells during follicular growth and progressively decreases with increased atresia^49,50^. In the present study, PCNA immunoreactivity in the irradiated group was lower than the control group in almost all stages of follicular development, suggesting that cell proliferation ceased in response to cellular damage caused by γ-radiation. Contrariwise, a significant increase in the percentage of PCNA positive cells was observed in granulosa cells of irradiated rats treated with either GEN or E2 as compared to the untreated irradiated group. These data support a recent study demonstrating the proliferative effect of GEN on rat uterus^51^. The proliferative effects of GEN or E2 could be attributed to increased AMH levels which could act as a guardian to control the balance between proliferation and differentiation of germ cells^52^. To this point, these results stated the antioxidant, antiapoptotic, and proliferative properties of GEN in attenuating radiation-induced ovarian injury. However, the molecular mechanisms modulating GEN radioprotective effects remain still unclear. To understand the underlying mechanisms, ER-β, FOXL2, and TGF-β gene expressions were assessed.

Phytoestrogens function as natural SERMs, depending on the tissue and the presence of co-regulator proteins^12^. Interestingly, GEN can bind both ER isoforms, although it binds ER-β with a 20-fold higher affinity compared with ER-α^53^. The antioxidant effects of GEN could be attributed to its estrogenic activity by acting on ER-β, with minimal side effects because of minimal interactions with ER-*α* compared with E2^54^. In the ovary, ERβ is the major receptor in granulosa cells, which promotes the proliferation of granulosa cells in early folliculogenesis^55^ and also follicular maturation from the early antral to preovulatory stages^56^. Moreover, it has been reported that antioxidant and antiapoptotic activities of GEN are selectively relative, at least in part, to ERβ-mediated signaling^54,57^. The present study demonstrated that both GEN and E2 treatment showed up-regulation of ER-β mRNA expression in ovaries of irradiated rats which may have a beneficial role in attenuating oxidative stress and apoptosis with enhancing granulosa cells proliferation. More upstream in the apoptotic cascade, ER-β has specific effects on Bax expression which may be related to the mitochondrial localization of ER-β^58^, suggesting a direct estrogenic effect on mitochondria function via ER-β activation. As previously mentioned, GEN binds ER-β with a 20-fold higher affinity compared with ER*α*. Therefore, the reduction of Bax, cytochrome c, and caspase 3 expressions by isoflavone GEN could be attributed to ER-β upregulation leading to disruption of apoptotic signaling by downregulating key pro-apoptotic factors in the cell death cascade. It is plausible that GEN exerts protective effects against γ-radiation-induced ovarian injury via enhancing ER-β expression.

Implication and regulation of ovarian follicles transitions include transcription factors TGF-β and FOXL2 which co-operatively modulate granulosa cells apoptosis and proliferation. TGF-β promotes ovarian development by activating the transition of primary follicles into the pre-antral and antral stages^11^. Although TGF-β is required for proper follicular development and female fertility^59^, TGF-β plays a role in mammalian granulosa cells apoptosis^60^. Recently, TGF-β signaling has been implicated in radiation-induced POF^61^, and is well established to negatively affect the secretion of E2^60^ which is confirmed by findings of the present study.

On the other hand, the FOXL2 gene encoding a forkhead transcription factor is involved in almost all stages of ovarian development and function^62^. The specific positive signals for FOXL2 transcripts suggest its functional importance in oocyte development, granulosa cell proliferation, and follicles differentiation^63^. Once expressed in pre-granulosa cells, FOXL2 is considered as one of the inhibitory signals for follicular activation that maintain primordial follicles in the dormant state thus preserving the follicular stock^64^. Accordingly, the germline mutation of FOXL2 is associated with POF^64^, which can be explained by the early depletion of the primordial follicle pool. This depletion is because of premature activation of almost all primordial follicles as a result of granulosa cell differentiation failure and leads to widespread follicular atresia. Consistent with the aforementioned studies, we observed that γ-radiation downregulates FOXL2 expression which may explain the depletion of primordial follicles and diminished granulosa cell proliferation. Subsequently, most of the oocytes and granulosa cells grow and undergo atresia due to the absence of follicular cell support, suggesting that granulosa cell function is not only required for oocyte growth but also to maintain follicular quiescence. Our results also support the previous reports regarding FOXL2^*lacZ*^ homozygous mutant ovaries which detected that general oocyte activation correlates with the absence of secondary follicles, leading to a complete depletion of the primordial follicle reserve pool^65^. Therefore, γ-radiation could provoke follicles depletion, induce apoptosis, and diminish proliferation through upregulating TGF-β and downregulating FOXL2 expressions. Treatment with GEN counteracts the effects of radiation leading to the preservation of primordial follicles stock and preventing follicular atresia. Inhibitory effects of GEN on TGF-β expression confirmed previous reports demonstrating the protective effects of GEN through blocking the TGF-β signaling pathway^66^. On the other hand, E2 administration although significantly increased FOXL2 expression, it could not affect TGF-β expression which could explain the ability of E2 to preserve the primordial follicle stock from radiation damage without affecting the antral and atretic follicle populations.

Although the current study was limited by exploring the modulatory role of GEN in an experimental model of radiation-induced POF, our findings shed light on the ovarian radioprotective approach of GEN and novel insights into its molecular targets. Therefore, GEN could be considered as a novel therapeutic modality for preserving ovarian function in female cancer survivors during radiotherapy. Nevertheless, further studies directed towards building preclinical and clinical models to evaluate the safety of GEN administration on female reproduction will help to further elucidate the beneficial effects of GEN. Noteworthy, the epigenetic role of GEN in the regulation of multiple genes and proteins involved in ovarian follicles transition and maturation should also be considered. Moreover, more studies should be conducted to explore the influence of GEN on reproductive capacity in terms of fecundability and fecundity.

In conclusion, the present study demonstrates that GEN protects against radiation-induced ovarian injury through decreasing histopathological alteration, restoring anti-oxidant capacity, enhancing granulosa cells proliferation, and ameliorating the intrinsic pathway of apoptosis. Comparing with E2, GEN is superior in preserving all stages of healthy follicles and diminishing the atretic follicle population. The mechanism underlying the promising effects of GEN over E2 could be through attenuation of the intrinsic pathway of apoptosis which could partly be mediated through upregulating ER-β expression. Moreover, GEN was able to increase FOXL2 expression with consequent downregulation of TGF-β expression leading to inhibition of ovarian follicles transition and subsequently hampers follicles atresia with preservation of primordial follicles stock. Therefore, GEN may constitute a novel therapeutic modality for safeguarding folliculogenesis and alleviating radiation-induced POF.

## Methods

### Chemicals and reagents

GEN was purchased from Winherb Medical Tech Co. Ltd. (China). Ethinyl estradiol (E2) was purchased from Nile Co. for Pharmaceutical and Chemical industries, Egypt. Dimethylsulfoxide (DMSO) was obtained from Sigma Chemical Co. (St. Louis, MO, USA). AMH ELISA assay kit was procured from Cusabio Biotech Co. (Wuhan, China). Estradiol ELISA kit was purchased from Monobind Inc. (Lake Forest, CA 92630, USA). Glutathione peroxidase (GPx) kit was attained from Randox Laboratories (UK). Reduced glutathione kit was purchased from Biodiagnostics Company (Cairo, Egypt). Purelink RNA isolation kit, SuperScript Reverse Transcription Kit, SYBR Green Master Mix kit, and Trizole reagent were purchased from Life Technologies Co. (Carlsbad, CA, USA). All other chemicals and solvents were of the highest grade commercially available.

### Animals

Three-week-old female Sprague-Dawley rats (weighing 30–40 g) were obtained from Nile Co. for Pharmaceutical and Chemical industries (Egypt). Rats were housed under standard laboratory conditions in specially prepared cages in a room with a 12-h light/dark cycles, at 25 ± 2 °C and 55 ± 5% relative humidity. Rats were kept on standard diet pellets (El-Nasr chemical company, Egypt) contained not less than 20% protein, 3.5% fat, 6.5% ash, 5% fiber and a vitamin mixture and allowed free access to drinking water ad libitum.

### Irradiation

Immature female rats were exposed to whole-body radiation using Cesium (^137^CS) source, Gamma Cell-40 biological irradiator, at the National center for radiation research and technology, Atomic energy authority, Cairo, Egypt. Animals were exposed to 3.2 Gy single dose γ-radiation at a dose rate of 0.48 Gy per min. The dose of γ-radiation which is sufficient to induce POF in rats was chosen according to previous studies^67,68^.

### Experimental design

A priori power analysis was conducted using GPower 3.0.10 software to estimate the sample size needed for the experiment and accordingly, the number of rats per group. Animals were divided randomly into five groups (20 animals per group) and treated for 10 days as follows:

Group 1 (Control): rats received 5 ml/kg of the GEN and E2 vehicle (DMSO and corn oil at a ratio of 1:10), once daily.

Group 2 (Irradiated): rats received 5 ml/kg of the vehicle, once daily. On the 7th day, rats were exposed to 3.2 Gy single dose γ-rays 1-h after vehicle administration.

Group 3 (GEN): rats were injected with GEN (5 mg/kg B.W, i.p.), once daily.

Group 4 (GEN/irradiated): rats were injected with GEN (5 mg/kg B.W, i.p.), once daily. On the 7th day, rats were exposed to 3.2 Gy single dose γ-rays 1-h after GEN treatment.

Group 5 (E2/irradiated): rats were injected with E2 (0.1 mg/kg B.W., s.c.), once daily. On the 7th day, rats were exposed to 3.2 Gy single dose γ-rays 1-h after E2 treatment.

The doses of GEN and E2 were chosen according to previously reported studies^41,69^. Animals' body weights were recorded daily until the sacrifice day. Twenty-four hours after the treatment had ended, blood samples were collected from the retro-orbital plexus and permitted to clot. The serum was separated by centrifugation at 5,000 rpm for 15 min and then kept frozen at − 80 °C. Thereafter, rats were sacrificed by cervical dislocation and ovarian tissues were isolated, washed with ice-cold saline, and weighed. After weighing the ovaries, some of which were stored at − 80 °C for biological assessment while others were fixed in a suitable buffer for histological and immunohistochemical examination.

### Assessment of circulating levels of anti-Müllerian hormone (AMH) and estradiol

In order to assess the follicular reserve, we measured serum AMH levels using the AMH ELISA kit. The intra- and inter-assay variation coefficients for AMH were found to be less than 15%. Serum estradiol was also measured using the estradiol ELISA kit. The intra- and inter-assay variation coefficients for estradiol were found to be less than 9% and 10%, respectively. The minimum detectable concentrations for AMH and E2 were 0.375 ng/ml and 3.6 pg/ml, respectively.

### Assessment of oxidative stress markers

Ovarian tissues were homogenized at 1:10 (w:v) in phosphate buffer saline (pH 7.4) with a homogenizer then, the supernatant was obtained by centrifugation at 10,000 rpm for 15 min. Reduced glutathione (GSH) levels were determined colorimetrically according to Beutler et al.^70^ method using GSH kit and the results were expressed as μg/mg wet tissue. Furthermore, glutathione peroxidase (GPx) activity was determined spectrophotometrically according to Paglia and Valentine^71^ method and the specific activity was expressed as U/g wet tissue.

### Histopathological examination

The ovaries were fixed in 10% neutral buffered formaldehyde overnight and then embedded in paraffin. Serial sections of 4 µm thick were deparaffinised with xylene and then, stained with hematoxylin and eosin for histological examination under a light microscope.

### Morphometric analysis of follicles population

Following histopathological examination, the fifth cut in all ovarian samples was used for counting follicles number per each follicle category and for evaluation of follicular development across six sections using a digital video camera mounted on a light microscope (CX21, Olympus, Tokyo, Japan). Follicles were classified according to their development stages into primordial, preantral, antral, and atretic as previously prescribed^72,73^.

### Immunohistochemical detection of ovarian proliferation cell nuclear antigen (PCNA) and apoptotic markers (cytochrome c and caspase-3)

Paraffin-embedded ovarian sections, 3 µm thick were dehydrated first in xylene and next in graded ethanol solutions. The slides were then blocked with 5% bovine serum albumin in Tris-buffered saline for 2 h. After that, immunohistochemical analyses were performed using the standard streptavidin–biotin–peroxidase procedure. The sections were incubated overnight at 4 °C with either a mouse anti-PCNA monoclonal antibody (Thermo Fisher Scientific, Cat. No. MS- 106-R7), a mouse anti-cytochrome c monoclonal antibody (Thermo Fisher Scientific, Cat. No. MS-1192-R7) or a rabbit anti-caspase 3 polyclonal antibody (Thermo Fisher Scientific, Cat. No. PA5-77887). After rinsing the sections comprehensively with Tris-buffered saline, they were incubated for 10–15 min with a biotinylated goat anti-rabbit secondary antibody, and afterward, the horseradish-peroxidase-conjugated streptavidin solution was added and incubated for 10–15 min at room temperature. Sections were then incubated for 5–10 min in 0.02% diaminobenzidine solution containing 0.01% H_2_O_2_^67^. Counterstaining was performed using hematoxylin, and the slides were examined under a light microscope. The digital colour images were registered using a light microscope (CX21, OLYMPUS, JAPAN) equipped with a camera AxioCam HRc (Carl Zeiss, Jena, Germany) and connected to a computer.

Positive staining was specified by intense reddish-brown staining of the cell nucleus/cytoplasm and negative staining was specified by light or diffuse staining of the cell nucleus/cytoplasm^74^. To estimate the percentage of proliferating cells, the number of PCNA positively-stained granulosa cells over the total number of granulosa cells was counted in seven high-power fields (40×) using a digital video camera, then, the percentage of PCNA positive cells was calculated. In contrast, the immunohistochemical quantification of cytochrome c and caspse-3 was carried out by measuring the optical density in seven high power fields of five rat ovaries using image analysis software (Image J, 1.46a, NIH, USA)^75^.

### Real-Time polymerase chain reaction (qRT-PCR) of Bax, Bcl-2, ER-β, FOXL-2, and TGF-β mRNA levels

Trizole reagents were used to extract total RNA from five whole ovaries per group using Tissue Lyser LT (Qiagen, Germany). RNAs were then purified using the Purelink RNA mini kit according to the manufacturer’s instructions. RNA purity and quantity were assessed by measuring the optical density at 260 nm using the NanoDrop spectrophotometer (Thermo Scientific). RNA purity and quantity were between (1.8–2.2) and (1,000–2,500 ng/μl), respectively. Roughly 1 μg of total RNA was reverse transcribed in 20 μl reaction volume using Super Script Reverse Transcription Kit. The PCR reaction was carried out in a reaction mixture of 25 μl total volume containing 2 μl of cDNA, 12.5 μl of SYBR Green Master Mix, and 20 μM of primers using an "Applied Biosystems Step One real-time" PCR instrument. The experimental run protocol used was as follows: initial denaturation for 10 min at 95 °C, followed by 45 amplification cycles of 95 °C for 15 s and combined annealing/extension for 1 min at 60 °C. The melting curve of each amplification was determined after PCR amplification to verify the amplification accuracy^76^.

The cycle threshold was determined thereafter and the relative quantification value of target genes was normalized to the level of glyceraldehydes-3-phosphate dehydrogenase (GAPDH) reference gene. The Ct values for the target genes were measured for each sample, and the relative transcript levels were calculated as *x* = 2^−∆∆ct^ in which ∆∆ct = ∆Treatment − ∆C where ∆Treatment = Ct_treatment_ − Ct_GAPDH_ and ∆C = Ct_control_ − Ct_GAPDH_^77^. The sequences of primers for each gene are illustrated in Table 3.

**Table 3:** Sequences of primers sets used for the analysis of gene expression.

Gene symbol	Oligo	Primer sequence
Bax	Forward	5′-GATCAGCTCGGGCACTTTAG-3′
	Reverse	5′-TGTTTGCTGATGGCAA CTTC-3′
Bcl-2	Forward	5′-AGGAT TGTGG CCTTC TTTGA GT-3′
	Reverse	5′-GCCG GTTC AGG TACT CAGT CAT-3′
ER-Beta	Forward	5′-TTGGTGTGAAGCA AGAT CACTAGAG-3′
	Reverse	5′-AACAGGGCTGGCACAACTG-3′
FOXL2	Forward	5′-TCGCTAAGTTCCCGTTCTAC-3'
	Reverse	5′-GTAATTGCCCTTCTCGAACA-3'
TGF-β	Forward	5′-ACT ACT ACG CCA AGG AGG TCA C-3′
	Reverse	5′-TGCTTG AAC TTG TCA TAG ATT TCG-3′
GAPDH	Forward	5′-TCCCTCAAGATTGTCAGCAA-3'
	Reverse	5′-AGATCCACAACGGATACATT-3'

*GAPDH* glyceraldehydes-3-phosphate dehydrogenase, *Bcl-2* B-cell lymphoma 2, *Bax* bcl-2-like protein 4, *FOXL2* forkhead box protein L2, *TGF-β* transforming growth factor beta.

### Statistical analysis

Data are presented as the mean ± SD. Conformity to normality of data distribution was tested with Kolmogorov–Smirnov (KS) test. Multiple comparisons were analyzed by a one-way ANOVA test followed by Tukey–Kramer as a post-hoc test^78^. The statistical significance criterion used was the 0.05 level of probability. Statistical analyses were carried out using Instat version 3 and GraphPad Prism version 5 software (ISI software, USA).

### Ethics statement

Animal care and all experimental protocols were approved and conducted in accordance ethical guidelines approved by Research Ethics Committee of Faculty of Pharmacy, Helwan University, Cairo, Egypt [Approval number: 001a-16].
